# RNA-guided DNA base damage repair via DNA polymerase-mediated nick translation

**DOI:** 10.1093/nar/gkac1178

**Published:** 2022-12-19

**Authors:** Pawlos S Tsegay, Daniela Hernandez, Fei Qu, Mustapha Olatunji, Yasir Mamun, Prem Chapagain, Yuan Liu

**Affiliations:** Biochemistry Ph.D. Program, Florida International University, Miami, FL, USA; Department of Chemistry and Biochemistry, Florida International University, Miami, FL, USA; Biochemistry Ph.D. Program, Florida International University, Miami, FL, USA; Biochemistry Ph.D. Program, Florida International University, Miami, FL, USA; Biochemistry Ph.D. Program, Florida International University, Miami, FL, USA; Department of Physics, Florida International University, Miami, FL, USA; Biomolecular Sciences Institute, Florida International University, Miami, FL, USA; Biochemistry Ph.D. Program, Florida International University, Miami, FL, USA; Department of Chemistry and Biochemistry, Florida International University, Miami, FL, USA; Biomolecular Sciences Institute, Florida International University, Miami, FL, USA

## Abstract

DNA repair is mediated by DNA synthesis guided by a DNA template. Recent studies have shown that DNA repair can also be accomplished by RNA-guided DNA synthesis. However, it remains unknown how RNA can guide DNA synthesis to repair DNA damage. In this study, we revealed the molecular mechanisms underlying RNA-guided DNA synthesis and base damage repair mediated by human repair DNA polymerases. We showed that pol β, pol κ, and pol ι predominantly synthesized one nucleotide, and pol η, pol ν, and pol θ synthesized multi-nucleotides during RNA-guided DNA base damage repair. The steady-state kinetics showed that pol η exhibited more efficient RNA-guided DNA synthesis than pol β. Using molecular dynamics simulation, we further revealed dynamic conformational changes of pol β and pol η and their structural basis to accommodate the RNA template and misoriented triphosphates of an incoming nucleotide. We demonstrated that RNA-guided base damage repair could be accomplished by the RNA-guided DNA strand-displacement synthesis and nick translation leading to nick ligation in a double-strand DNA region. Our study revealed a novel RNA-guided base damage repair pathway during transcription and DNA replication.

## INTRODUCTION

Genome integrity and stability must be maintained to allow the faithful transfer of genetic information from parental to daughter cells (1,2). However, genomic DNA is constantly damaged by endogenous and environmental stressors (3,4). Major DNA damage includes DNA base damage, mismatches, DNA adducts, thymine dimers and single- and double-strand breaks (4). Among them, DNA base damage is the most common form and is generated at a rate of ∼10^4^ base lesions per cell per day (3). If not repaired, DNA base lesions can be converted into single-strand DNA breaks, ultimately double-strand breaks (DSBs), causing chromosomal breakage, DNA recombination and rearrangement, and cell death (5).

To combat the adverse effects of DNA damage, cells have evolved several DNA repair pathways to remove various DNA damage (4). A critical step of DNA repair is DNA synthesis performed by DNA polymerases. DNA synthesis is essential to fill in gaps, generate nicks, and allow the ligation of nicks and completion of repair. Specific DNA polymerases are employed to repair different types of DNA damage using DNA as a template in cells. In some cases, multiple DNA repair polymerases cooperate to accomplish DNA damage repair (6). However, several studies have shown that eukaryotic DNA polymerase, including human pol γ (7,8) and yeast pol δ and pol α, can also synthesize DNA using RNA as a template (9), although RNA usually acts as a template for reverse transcriptases to synthesize cDNA during the reverse transcription of retroviruses and retrotransposons (10) and telomere synthesis (11,12). Since RNA transcripts are synthesized from their DNA templates and can transiently cause the formation of an RNA–DNA hybrid, it is conceivable that cells may exploit the sequence homology of RNA to repair damaged DNA templates. This possibility is supported by several recent studies that implicate the role of RNA in guiding DNA repair (13–16). This has been further supported by the studies showing that RNA can indirectly mediate DNA recombination through a cDNA molecule in yeast (17,18), and that RNA transcripts can facilitate precise DNA repair (19). RNA-guided DNA repair is also implicated in mammalian cells. It is found that long interspersed elements (LINE-1) retrotransposons can employ retrotranscription to repair DNA strand breaks and integrate themselves at the damaged sites (20). It is also found that DNA damage can induce long non-coding RNAs (dilncRNAs) and small DNA damage response RNAs (DDRNAs) that are subsequently recruited to DSB sites to promote DNA repair (21,22). Moreover, it has been found that dilncRNAs can form RNA–DNA hybrids at DSBs, leading to the recruitment of BRCA1, BRCA2, RAD51 and NBS1 to DSBs for DSB repair (13,14,16). Most recently, a study has demonstrated that the human translesion polymerase, pol θ exhibits reverse transcriptase activity to mediate non-homologous end joining during repair of DSBs (23). Also, RNA can be involved in UV-induced DNA damage repair through the modified RNA base, N^6^-methyladenosine (m^6^A) (15), which can recruit pol κ to UV damaged sites, thereby promoting nucleotide excision repair and translesion synthesis (15). A recent study further demonstrates that m^6^A on mRNA and lncRNA can be induced by DSB breaks via the activation of N^6^-adenosine-methyltransferase like 3 (METLL3), to recruit RAD51 and BRCA1 to DNA damage sites and promoting DSB repair (24). All the studies suggest that RNA can mediate DNA repair by serving as a template for DNA synthesis and recruiting DNA repair proteins.

RNA-guided DNA repair can occur on RNA–DNA hybrids formed during DNA lagging strand synthesis and gene transcription. Studies have shown that DNA damage accumulates on RNA–DNA hybrids (13,15). Also, RNA–DNA hybrids may promote the accumulation of DNA damage and genome instability (25) through R-loops, the hotspots of DNA damage (26). We have recently shown a unique mechanism by which DNA base excision repair (BER) removes a DNA base lesion on the non-template DNA strand of a CAG repeat R-loop leading to its resolution and repeat deletion (27). However, it remains unknown if DNA base damage on the template strand of RNA–DNA hybrids can be repaired directly using an RNA as a template. Since more studies have shown that RNA can guide DNA synthesis and is involved in DNA repair (7–9,15,16,23), we hypothesize that RNA can serve as a template to guide DNA synthesis to mediate base damage repair on RNA–DNA hybrids. To test the hypothesis, we initially characterized RNA-guided DNA synthesis activity of replication and repair DNA polymerases on different DNA repair intermediates with an RNA template. We then examined the enzymatic activities of key BER enzymes on the RNA–DNA hybrid intermediates. We found that human replicative DNA polymerases, pol δ and pol ϵ failed to synthesize DNA with an RNA template. Human pol β, pol ι, and the translesion DNA polymerase, pol κ only performed one nucleotide gap-filling synthesis. The translesion DNA polymerases, pol η, pol θ and pol ν performed one nucleotide gap-filling synthesis and strand-displacement synthesis with an RNA template. We showed that the DNA nick opposite to the RNA template had to be translated through the strand-displacement synthesis to a double-strand DNA region for ligation. Our results indicated that the completion of RNA-guided DNA base lesion repair was accomplished through a nick translation demonstrating a unique RNA-guided base damage repair pathway. Steady-state kinetics showed that the efficiency of RNA-guided DNA synthesis by pol β was significantly reduced compared with DNA-guided synthesis. However, pol η exhibited the same catalytic efficiency of synthesizing DNA with the RNA template as the DNA template. Using the molecular dynamics simulation, we further revealed the molecular basis underlying the RNA-guided DNA synthesis.

## MATERIALS AND METHODS

### Materials

Oligonucleotides were synthesized by Eurofins Genomics (Louisville, KY, USA). Radionucleotide, ^32^P-ATP (6000 μCi/mmol) was purchased from PerkinElmer Inc. (Boston, MA, USA). Micro Bio-Spin 6 chromatography columns were from Bio-Rad Laboratories (Hercules, CA, USA). Pol β and DNA ligase I (LIG I) were expressed and purified as described previously (28). Human pol η, pol δ catalytic unit (125 kD), pol θ, and pol ν were provided by Dr. Wei Yang from the National Institute of Diabetes and Digestive and Kidney Diseases/National Institutes of Health as described previously (29). Human pol κ, pol ι and pol λ were purchased from ENZYMAX (Lexington, KY, USA). M-MLV reverse transcriptase was from Promega Corporation (Madison, WI, USA). All other standard chemical reagents were from Sigma–Aldrich (St. Louis, MO, USA) and ThermoFisher Scientific (Pittsburgh, PA, USA).

### Oligonucleotide substrates

An open template substrate with a DNA primer and an RNA or DNA template was designed to mimic the substrate to test the RNA- and DNA-guided DNA synthesis by translesion DNA polymerases. Substrates containing one nucleotide gap were designed to test RNA- and DNA-guided gap-filling synthesis on different BER intermediates. The open template substrates for testing RNA- and DNA-guided DNA synthesis were constructed by annealing a 19 nt-upstream DNA primer with a 36 nt-RNA or 36 nt-DNA template with a random sequence or a 30 nt-RNA template containing the RNA sequence of COVID-19 spike protein (Supplementary Table S1). The open template substrate for testing RNA-guided DNA synthesis on hepatitis C virus (HCV) was constructed by annealing an 18 nt-upstream DNA primer with a 30 nt RNA or DNA template containing HCV RNA sequence (Supplementary Table S1). The 1 nt gap substrates for testing RNA- and DNA-guided gap-filling synthesis were constructed by annealing the 19 nt-upstream DNA primer and 16 nt-downstream DNA primer containing either a 5′-phosphate or 5′-phosphorylated tetrahydrofuran (THF) residue with the 36 nt-RNA or 36 nt-DNA template containing dC opposite to the 1 nt gap (Supplementary Table S1). The nick substrate for the reconstituted BER was constructed by annealing the 19 nt DNA primer with a 17 nt 5′-phosphorylated downstream DNA primer and the 36 nt-RNA template. The substrates with a nick at various locations relative to an RNA template were constructed with the 19 nt RNA annealed with DNA making 3 nt, 6 nt and 9 nt annealed with a DNA strand, respectively. All substrates were assembled by annealing the upstream with the template strand with a molar ratio at 1:3 or by annealing the upstream and downstream primers with the template strands at a molar ratio of 1:2:3. The substrates were radiolabeled at the 5′-end of the upstream DNA primer.

### Enzymatic activity assays

RNA-templated DNA synthesis activities of various DNA polymerases were measured by incubating 25 nM substrates with fixed or increased concentrations of the DNA polymerases in the presence of 50 μM dNTPs at 37°C for 30 min in reaction mixture (10 μl) containing the BER reaction buffer with 50 mM Tris–HCl, pH 7.5, 50 mM KCl, 5 mM Mg^2+^, 0.1 mM EDTA, 0.1 mg/ml bovine serum albumin, and 0.01% Nonidet NP-40. The buffer system was chosen because it provided the optimal condition for DNA polymerases to achieve the high efficiency of DNA synthesis, as previously reported (30–37). RNA-guided reconstituted BER reactions were performed by incubating 25 nM substrates containing a native abasic site (AP site) generated from a uracil using 5 U bacterial uracil DNA glycosylase (UDG) (New England BioLabs, Ipswich, MA) or a reduced AP site, tetrahydrofuran (THF) residue with 25 nM APE1, 25 nM pol β or pol η, 25 nM LIG I in the absence (for the native AP site) or presence (for the reduced AP site) of FEN1 in the BER reaction buffer containing 2 mM ATP. RNA-templated DNA ligation by LIG I was examined in the BER reaction buffer containing 2 mM ATP in the presence of 25 nM substrates with a nick at different locations relative to the RNA template. Substrates and products were separated by 15% urea-denaturing polyacrylamide gel and detected by Pharos FX Plus PhosphorImager (Bio-Rad Laboratories, Hercules, CA). All experiments were repeated independently at least three times.

### Steady-state kinetics of RNA-templated DNA synthesis

The steady-state kinetics of DNA synthesis by pol β and pol η was determined using a fixed concentration of the DNA polymerases with increasing concentrations of the DNA–RNA or DNA–DNA hybrid substrates in the presence of 50 μM dG for the 1 nt gap substrate and 50 μM dNTPs or increasing concentrations of a deoxyribonucleotide triphosphate (25–500 μM) for the 1 nt gap substrates and open template substrate (50 nM). The enzymes were incubated with the substrates at 37°C at different time intervals (0 to 15 min) in reaction mixture (10 μl) containing BER buffer with 50 mM Tris–HCl, pH 7.5, 50 mM KCl, 5 mM Mg^2+^, 0.1 mM EDTA, 0.1 mg/ml bovine serum albumin, and 0.01% Nonidet NP-40. The reactions were stopped using 2× stopping buffer containing 95% deionized formamide and 10 mM EDTA, 0.05% (w/v) bromophenol blue, Sigma-Aldrich (St. Louis, MO) and 0.05% (w/v) xylene cyanol, Sigma-Aldrich (St. Louis, MO) followed by incubation at 95°C for 5 min. Substrates and products were separated by 15% urea-denaturing polyacrylamide gel and detected by Pharos FX Plus PhosphorImager (Bio-Rad Laboratories, Hercules, CA). The apparent Michaelis–Menten constants, *V*_max_, *K*_m_, and *k*_cat_ values were calculated using the Enzyme Kinetics Module of the Prism-GraphPad software, version 6.03.

### Molecular dynamics simulations

The X-ray crystal structures of pol β (PDB ID 5TBB) (38) and pol η (PDB ID 4J9N) (39) were taken from the protein data bank. The complex structures suit our objectives as they illustrate the interaction of pol β and pol η with a 1 nt-gap and open template substrate. For the incoming nucleotide in pol η, the modified guanosine XG4 in the crystal structure was replaced by dGTP, and the incoming nucleotide dCTP for pol β was docked to the position using AutoDock Vina (40). The two systems of pol β and pol η with DNA templates were prepared for molecular dynamics simulation using the solution builder tool in CHARMM-GUI (40–42). The RNA template strand was created based on the existing DNA template in the crystal structures of the polymerase-DNA complexes because no crystal structures with the RNA template are available. For this, the Charmm36m force-field was employed to treat the deoxyribonucleotides as ribonucleotides. Specifically, deoxynucleotides in the DNA template were read by the psfgen without the DEOX patch of the Charmm36m force-field, converting the coordinates of the existing DNA template into the RNA template. Consequently, the psfgen plugin of VMD used the existing coordinates of DNA to add the OH group to the deoxyribose and removed the methyl group from T, converting it to U. In this way, the base-paired RNA–DNA hybrid structure was modeled without perturbing the original structure, but with relaxation to appropriate RNA–DNA hybrid structure through Molecular Dynamics Simulation. The structures were solvated in a cubic box with TIP3P water model and 0.15 M NaCl. Two additional systems with RNA templates were prepared by converting the DNA template sequences to RNA using psfgen function in Visual Molecular Dynamics (VMD 1.9.3) (43). All four systems were simulated with Charmm36m force field (44,45) using the GPU version of NAMD 2.14 (46). The systems were minimized for 10 000 steps and equilibrated for 250 ps at 303 K and 1 atm pressure. The temperature was kept constant by using Langevin temperature coupling with a damping coefficient of 1/ps, and the pressure was kept constant using a Nose − Hoover Langevin piston (47) with a 50 fs period and 25 fs decay. The Particle Mesh Ewald method (PME) (48) was used for long-range electrostatic interactions with periodic boundary conditions. All covalent bonds with hydrogen atoms were constrained by ShakeH (49). For each system, a 200 ns unconstrained production run was performed with 2 fs/step. The trajectories were analyzed with VMD.

## RESULTS

### Repair and translesion DNA polymerases can perform DNA synthesis with an RNA template

**Figure 1.**
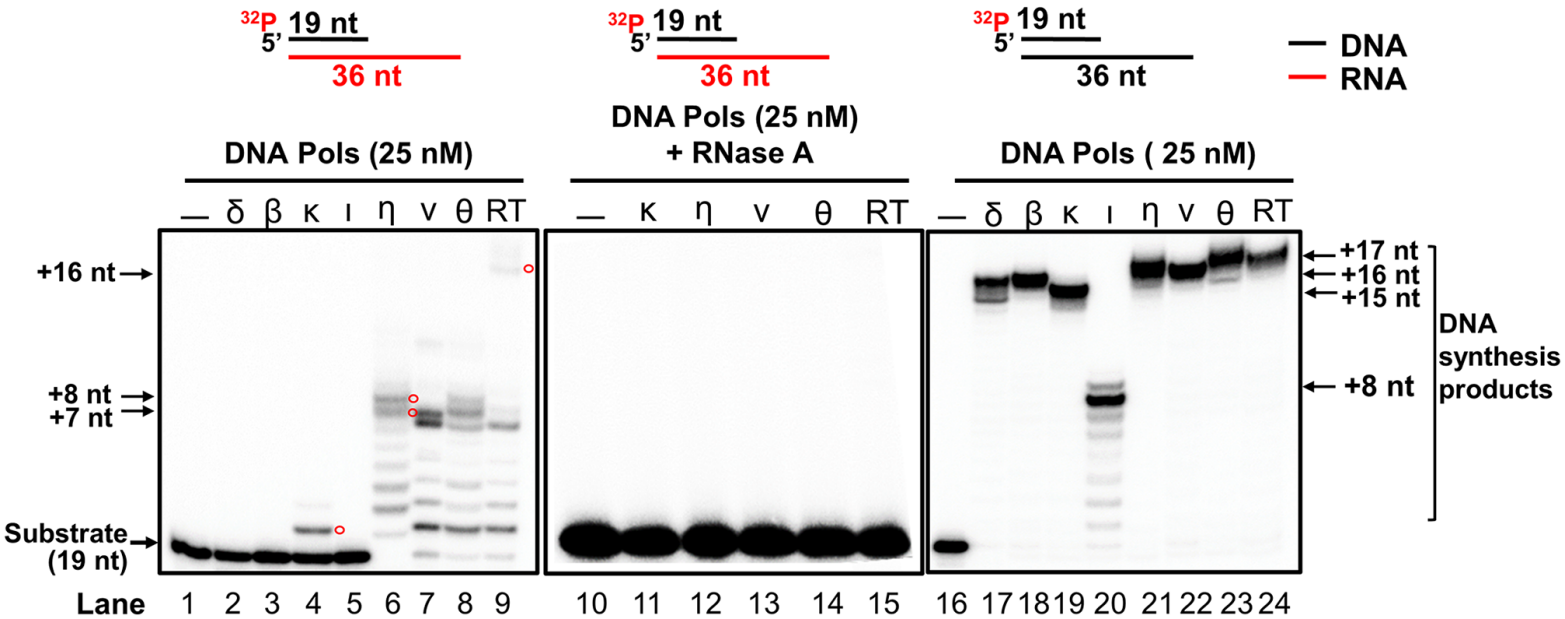
RNA- and DNA-templated DNA synthesis by DNA polymerases. RNA- and DNA-templated DNA synthesis by DNA polymerases was determined using a substrate (25 nM) constructed by annealing a DNA primer to a RNA or DNA template. Lanes 2–8 represent RNA-templated DNA synthesis by 25 nM of DNA polymerases. Lanes 11–14 represent RNA-templated DNA synthesis by 25 nM of DNA polymerases in the presence of RNase A (10 nM). Lanes 17–23 represent DNA-templated DNA synthesis by 25 nM of DNA polymerases. Lane 9, 15 and 24 represent RT synthesis. Lanes 1, 10 and 16 represent substrate only. All experiments were performed at least in triplicate.

**Figure 2.**
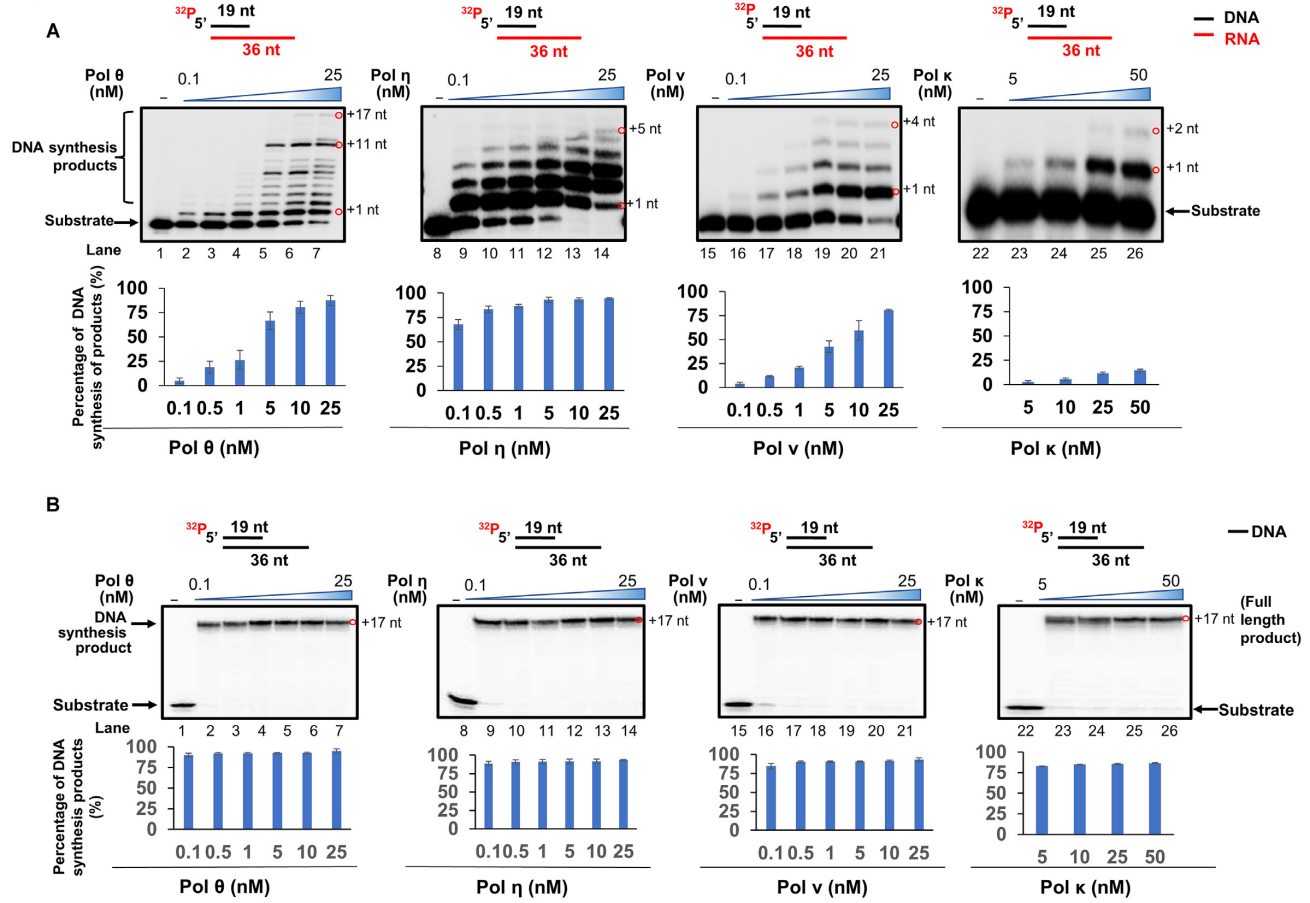
RNA-templated DNA synthesis on an open template substrate by DNA polymerases. RNA-templated DNA synthesis (**A**) and DNA-templated synthesis (**B**) by DNA polymerases on the open template substrates was determined by incubating an open template substrate containing an RNA template or DNA template and a DNA primer (25 nM) with a fixed concentration of dNTPs (50 μM) and increasing concentrations of DNA polymerases (0.1, 0.5, 1, 5, 10 and 25 nM for pol θ, pol η and pol ν and 5, 10, 25 and 50 nM for pol κ) at 37°C for 30 min. Lanes 2–7 represent DNA synthesis by pol θ. Lanes 9–14 represent DNA synthesis by pol η. Lanes 16–21 represent the DNA synthesis by pol ν. Lanes 23–26 represent pol κ DNA synthesis. Lanes 1, 8, 15 and 22 represent substrate only. The synthesized DNA products are indicated by red circles with their estimated sizes. All experiments were performed at least in triplicate.

DNA polymerases play an essential role in DNA replication and repair to maintain genome integrity and stability. However, repair DNA polymerases can exhibit structural flexibility to tolerate and bypass damaged nucleotides. Thus, it is possible that repair DNA polymerases may accommodate the effects of sugar pucker in an RNA template to perform DNA synthesis to aid DNA damage repair. To test this, we initially examined the DNA synthesis activity of replication, repair, and translesion DNA polymerases, pol δ, pol β, pol ι, pol κ, pol η, pol ν and pol θ using an open template substrate containing a DNA primer annealed with an RNA or DNA template (Figure 1). The results showed that pol δ, pol β and pol ι failed to perform DNA synthesis with the open RNA template (Figure 1, lanes 2–3 and lane 5). Pol δ and pol β exhibited efficient 16 nt-17 nt insertion on the open DNA template (Figure 1, lanes 17–18). On the other hand, pol κ, pol η, pol ν, and pol θ readily performed DNA synthesis on the open DNA template leading to 16 nt-17 nt insertion (Figure 1, lane 19 and lanes 21–23). Pol ι only inserted 8 nt on the DNA template (Figure 1, lane 20), whereas RT synthesized 17 nt DNA to the end of the DNA template leading to the 36 nt full-length product (Figure 1, lane 24). The results further suggest that the DNA synthesis of replicative and repair DNA polymerases were inhibited by the open RNA template. However, translesion DNA polymerases managed to accommodate the RNA template to synthesize DNA. We then tested if the DNA synthesis products specifically resulted from the RNA template by detecting the DNA synthesis in the presence of RNase A (Figure 1, lanes 11–15). We found that no DNA synthesis products were generated in the presence of RNase A indicating that the DNA synthesis was RNA template dependent. We then examined the RNA-templated DNA synthesis on the open template substrate by translesion DNA polymerases (Figure 2A). The results indicated that pol η, pol ν, and pol θ performed distributive DNA synthesis on the RNA template at the concentrations ranging from 0.1 to 25 nM (Figure 2A, lanes 2–7, 9–14, 16–21). However, pol κ at 5–50 nM mainly synthesized one nucleotide (Figure 2A, lanes 23–26). For the open RNA template, pol θ exhibited more efficient DNA synthesis than other DNA polymerases by inserting 1 nt-17 nt (Figure 2A, lanes 5–6). Pol θ, pol η, pol ν, and pol κ all exhibited efficient 17 nt insertion on the open DNA template leading to the formation of the 36 nt full-length product (Figure 2B, lanes 2–7, 9–14, 16–21, 23–26). Similar results were obtained with the DNA synthesis by pol η, pol ν, and pol θ on the open template substrates containing the RNA sequences of COVID-19 spike protein and hepatitis C virus RNA (Supplementary Figure S1). This indicates that the RNA-guided DNA synthesis by the translesion DNA polymerases is RNA sequence-independent and can occur on viral RNA templates.

### Repair and translesion DNA polymerases can perform RNA-guided gap-filling and strand-displacement synthesis during DNA base damage repair

**Figure 3.**
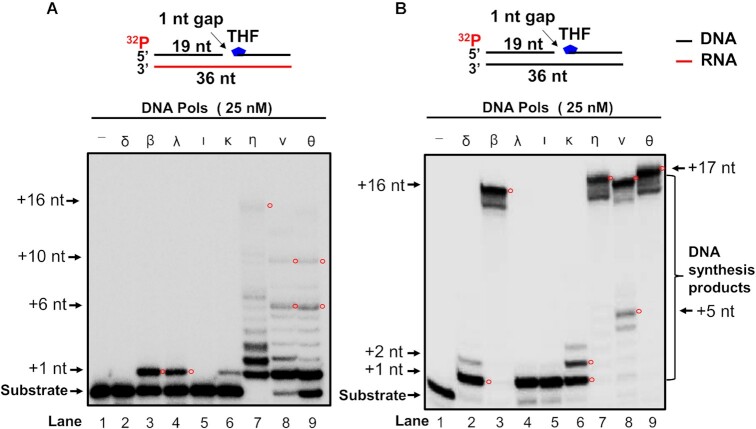
RNA-templated DNA synthesis at 1 nt gap with a sugar phosphate residue by DNA polymerases. RNA-templated DNA synthesis (**A**) and DNA-templated DNA synthesis (**B**) by various DNA polymerases was determined by incubating the RNA-templated or DNA-templated 1 nt gap substrate containing a 5′-phosphorylated THF residue that represents a sugar phosphate in the downstream primer (25 nM) with 25 nM DNA polymerases in the presence of 50 μM dNTPs at 37°C for 30 min. Lane 1 represents substrate only. Lanes 2–9 represent the reactions with 25 nM pol δ, pol β, pol λ, pol ι, pol κ, pol η, pol ν and pol θ, respectively. The synthesized DNA products are indicated by red circles with their estimated sizes. The experiments were performed in triplicate.

**Figure 4.**
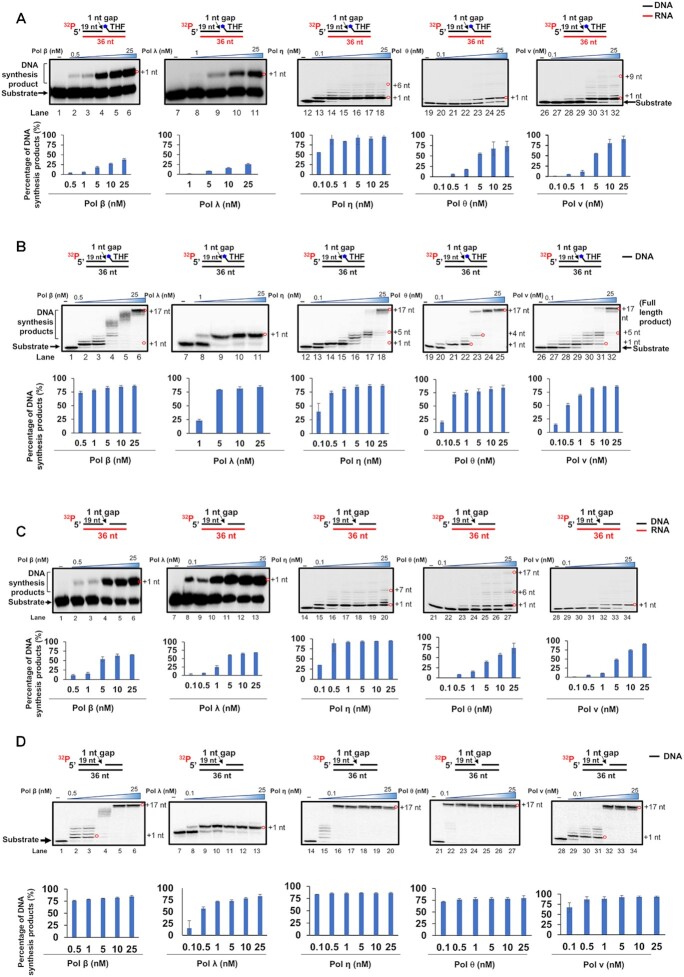
RNA-templated DNA synthesis with a 1 nt gap substrate with or without a THF residue by DNA polymerases. RNA-templated DNA synthesis (**A**) and (**C**) and DNA-templated DNA synthesis (**B**) and (**D**) by various DNA polymerases on a 1 nt-gap substrate (25 nM) with or without a THF residue on the downstream primer was examined in the presence of a fixed concentration of dNTPs (50 μM). Increasing concentrations of DNA polymerases (0.5, 1, 5, 10 and 25 nM for pol β, 1, 5, 10 and 25 nM for pol λ, and 0.1, 0.5, 1, 5, 10 and 25 nM for pol η, pol θ and pol ν) was incubated with 25 nM RNA-templated 1 nt gap substrate with THF (A) or the DNA-templated 1 nt gap substrate with THF (**B**). Lanes 2–6 represent the DNA synthesis by pol β. Lanes 8–11 represent the DNA synthesis by pol λ. Lanes 13–18 represent DNA synthesis by pol η. Lanes 20–25 represent DNA synthesis by pol θ. Lanes 27–32 represent DNA synthesis by pol ν. Lanes 1, 7, 12, 19 and 26 represent substrate only. RNA-templated DNA synthesis (**C**) and DNA-templated DNA synthesis (**D**) by pol β, pol λ, pol η, pol θ, and pol ν on a 1 nt-gap substrate without THF residue (25 nM) was examined in the presence of increasing concentrations of DNA polymerases (0.5, 1, 5, 10, and 25 nM for pol β, 0.1, 0.5, 1,5, 10 and 25 nM for pol λ, pol η, pol θ, and pol ν) and a fixed concentration of dNTPs (50 μM). Lanes 2–6 represent the DNA synthesis by pol β. Lanes 8–13 represent the DNA synthesis by pol λ. Lanes 15–20 represent the DNA synthesis by pol η. Lanes 22–27 represent the DNA synthesis by pol θ. Lanes 29–34 represent the DNA synthesis by pol ν. Lane 1, 7, 14, 21 and 28 represent substrate only. The synthesized DNA products are indicated by red circles with their estimated sizes. All experiments were performed in triplicate.

We then ask if the DNA polymerases can mediate RNA-templated BER. We tested this by characterizing RNA-templated DNA synthesis on the 1 nt-gap substrate with or without a deoxyribose phosphate (dRP) residue, which was represented by a 5′-phosphorylated THF residue (Figures 3 and 4). The substrates mimic the BER intermediates before and after the sugar phosphate residue is removed by pol β dRP lyase activity. We found that pol δ and pol ι failed to synthesize DNA on the RNA-templated 1 nt gap-THF substrate (Figure 3A, lanes 2 and 5). However, pol δ and pol ι, along with pol λ predominantly filled in 1 nt gap on the DNA-templated 1 nt gap-THF substrate (Figure 3B, lanes 2, and 4–5). Pol κ mainly inserted 1–2 nt on the substrate (Figure 3B, lane 6). In contrast, pol β, pol λ, pol κ, pol η, pol ν, and pol θ exhibited the RNA-templated DNA synthesis on the gapped THF substrate (Figure 3A, lanes 3–4, lanes 6–9, and Figure 4A). Pol β and pol λ only synthesized one nucleotide on the 1 nt gap substrate with or without THF (Figure 3A, lanes 3–4, Figure 4A, lanes 2–6 and lanes 8–11, Figure 4C, lanes 2–6 and lanes 8–13). Pol β at 0.5 -25 nM resulted in up to 25% of the one nucleotide gap-filling product with the substrate containing the THF residue (Figure 4A, lanes 2–6 and bar chart), whereas the same concentrations of the enzyme generated up to 60% synthesis product from the substrate without THF (Figure 4C, lanes 2–6 and bar chart). Pol λ exhibited comparable gap-filling synthesis with pol β on the substrates (Figure 4A, lanes 8–11 and Figure 4C, lanes 8–13, and bar charts). The polymerase at 0.1 -25 nM only generated up to 25% of product from the substrate with a THF residue (Figure 4A, lanes 8–11, bar chart). However, it generated up to 60% of products from the substrates without THF (Figure 4C, lanes 8–13, bar charts). Our results further demonstrated that the translesion DNA polymerases, pol η, pol ν, and pol θ predominantly synthesized one nucleotide on the RNA-templated gap substrates while they managed to continue to synthesize DNA and displaced the downstream strand (Figure 4A, lanes 13–18, 20–25 and 27–32, Figure 4C, lanes 15–20, 22–27 and 29–34). Increasing concentrations of the polymerases (0.1–25 nM) resulted in up to 80% of DNA synthesis products (Figure 4A and 4C, bar charts). The results indicated that pol β and pol λ performed 1 nt gap-filling synthesis, and pol η, pol ν, and pol θ performed both 1 nt gap-filling and strand-displacement synthesis on the RNA-templated substrates. For the DNA-templated 1 nt gap-THF substrate, pol β, pol η, pol ν, and pol θ performed efficient gap-filling and strand-displacement synthesis resulting in 1–17 nt insertion (Figure 3B, lane 3 and lanes 7–9, Figure 4B, lanes 2–6, 13–18, 20–25 and 27–32 and bar charts). Our results also showed that pol β, pol λ, pol η, pol θ, and pol ν exhibited efficient DNA synthesis on the DNA-templated 1 nt gap substrate (Figure 4D, lanes 2–6, 8–13, 15–20, 22–27 and 29–34 and bar charts). In addition, our results showed that pol θ performed more efficient DNA synthesis on the DNA-templated gapped substrates than other polymerases leading to the 36 nt full-length DNA synthesis product (Figure 4B, lanes 20–25 and 4D lanes 22–27).

However, we found that pol β failed to synthesize DNA on the nick substrate with the RNA template (Supplementary Figure S2, panel A). The results further indicated that the RNA-guided DNA synthesis by both pol β and pol λ was inhibited by the presence of THF (compare Figure 4A, lanes 2–6 and lanes 8–11 with Figure 4C, lanes 2–6 and lanes 8–13) suggesting that the 5′-dRP residue needs to be removed by the dRP lyase activity to facilitate the RNA-templated DNA synthesis. In contrast, Pol η generated strand-displacement synthesis products on the nick substrate (Supplementary Figure S2, panel B). It synthesized DNA more efficiently on the gapped DNA template substrates than other DNA polymerases leading to the generation of the 36 nt full-length DNA synthesis product (Figure 4B and 4D).

### The steady-state kinetics of RNA-guided DNA synthesis by pol β and pol η

We then used enzyme kinetics and molecular dynamic simulation to further explore the molecular and structural basis of RNA-templated DNA synthesis by pol β and pol η. These DNA polymerases were chosen because they represent a repair and translesion DNA polymerase, respectively. A high-resolution crystal structure is available for the polymerase. To compare the catalytic efficiency of RNA-templated DNA synthesis by pol β and pol η on the substrates containing 1 nt-gap and open template substrates, we performed the steady-state kinetics to determine the rate of RNA-templated DNA synthesis by the polymerases in the presence of various concentrations of dGTP (Table 1 and Table 2). The results indicated that the *K*_m_, *V*_max_, and *k*_cat_ of pol β for the 1 nt gap RNA template substrate without THF was 31.4 ± 0.1 × 10^−2^ μM, 12.0 ± 2.4 × 10^−11^ M s^−^^1^ and 1.2 × 10^−2^ s^−1^, respectively. For the substrate with THF, *K*_m_, *V*_max_, and *k*_cat_ were 33.5± 0.3 × 10^−2^ μM, 8.0 ± 0.4 × 10^−11^ M s^−1^ and 0.8 × 10^−2^ s^−1^, respectively (Table 1). The *k*_cat_/*K*_m_ of pol β on the substrate without THF was 1.6-fold higher than that with THF (Table 1). With the 1 nt-gap DNA template substrate, pol β exhibited *K*_m_ of 13.5 ± 0.1 × 10^−2^ μM, *V*_max_ of 7.0 ± 0.4 × 10^−11^ M s^−1^, and *k*_cat_ of 1.2 × 10^−2^ s^−1^. The catalytic efficiency is about 136-fold of that with the RNA template substrate (Table 1). Pol η exhibited *K*_m_, *V*_max_, and *k*_cat_ of 5.8 ± 0.9 × 10^−2^ μM, 13.2 ± 0.4 × 10^−11^ M s^−1^, and 13.2 × 10^−2^ s^−1^ for the 1 nt gap RNA template substrate without THF and 4.1 ± 1.3 × 10^−2^ μM, 15.6 ± 1.4 × 10^−11^ M s^−1^, and 15.6 × 10^−2^ s^−1^ for the substrate with THF (Table 2). The *k*_cat_/*K*_m_ of pol η for the gap substrate without and with THF was 22.70 × 10^5^ M^−1^ s^−1^ and 38 × 10^5^ M^−1^ s^−1^. Pol η catalytic efficiency for the open RNA template substrate was 31.70 × 10^5^ M^−1^ s^−1^. Its catalytic efficiency for 1 nt gap DNA template substrate was 22.50 × 10^5^ M^−1^ s^−1^, which was the same as the RNA template substrate (22.70 × 10^5^ M^−1^ s^−1^) (Table 2). Also, Pol η exhibited the same catalytic efficiency of 33.3 × 10^5^ M^−1^ s^−1^ for the DNA open template substrate as the one with the RNA template (31.70 × 10^5^ M^−1^ s^−1^) (Table 2). In contrast to pol β, the catalytic efficiency of pol η for the substrate with THF was 1.7-fold higher than the substrate without THF (Table 2). The catalytic efficiency of pol η for the substrate without and with THF was 60-fold and 159-fold of that of pol β, respectively. The catalytic efficiency of pol η for RNA open template was about 1.4-fold higher than that of the 1 nt gap substrate (Table 2). The results indicate that RNA-templated pol β gap-filling DNA synthesis was much less efficient than that of pol η. The pol β DNA synthesis was slightly inhibited by the 5′-deoxyribose phosphate. In contrast, the sugar phosphate residue slightly stimulated the RNA-guided gap-filling synthesis by Pol η. Pol η exhibited the same catalytic efficiency with RNA-templated DNA synthesis with the 1 nt-gap and the open-template substrates. Our results also showed that pol β exhibited 2.3-fold higher catalytic efficiency than pol η on the 1 nt-gap DNA substrate (Tables 1 and 2). This indicated that pol β preferred a DNA template over an RNA template, whereas pol η showed no preference for a DNA over an RNA template.

**Table 1.** Steady-state kinetics of RNA-guided DNA synthesis by pol β

Substrate	*K*_M_ (10^−2^ μM)	*V*_max_ (10^−11^ M s^−1^)	*k*_cat_ (10^−2^ s^−1^)	*k*_cat_ /*K*_M_ (× 10^5^ M^−1^ s^−1^)
1 nt gap-RNA template	31.4 ± 1.3	12.0 ± 2.4	1.2	0.38
1 nt gap-THF-RNA template	33.5 ± 0.3	8.0 ± 0.4	0.8	0.24
1 nt gap-DNA template	13.5 ± 0.1	7.0 ± 0.4	70.0	51.8

The kinetic experiment for table I and table II performed by varying the concentration of dGTP. All results are from at least three independent experiments.

**Table 2.** Steady-state kinetics of RNA-guided DNA synthesis by pol η

Substrate	*K*_M_ (10^−2^ μM)	*V*_max_ (10^−11^ M s^−1^)	*k*_cat_ (10^−2^s^−1^)	*k*_cat_ /*K*_M_ (× 10^5^ M^−1^ s^−1^)
1 nt gap-RNA template	5.8 ± 0.9	13.2 ± 0.4	13.2	22.70
1 nt gap-THF-RNA template	4.1 ± 1.3	15.6 ± 1.4	15.6	38.05
Open Template-RNA	0.4 ± 0.1	12.7 ± 0.4	12.7	31.70
1 nt gap-DNA template	14.8 ± 5.9	3.3 ± 1.3	33.3	22.50
Open template-DNA	12.0 ± 1.7	19.9 ± 1.1	39.9	33.30

### The structural basis underlying RNA-guided DNA synthesis by pol β and pol η revealed by molecular dynamics simulation

**Figure 5.**
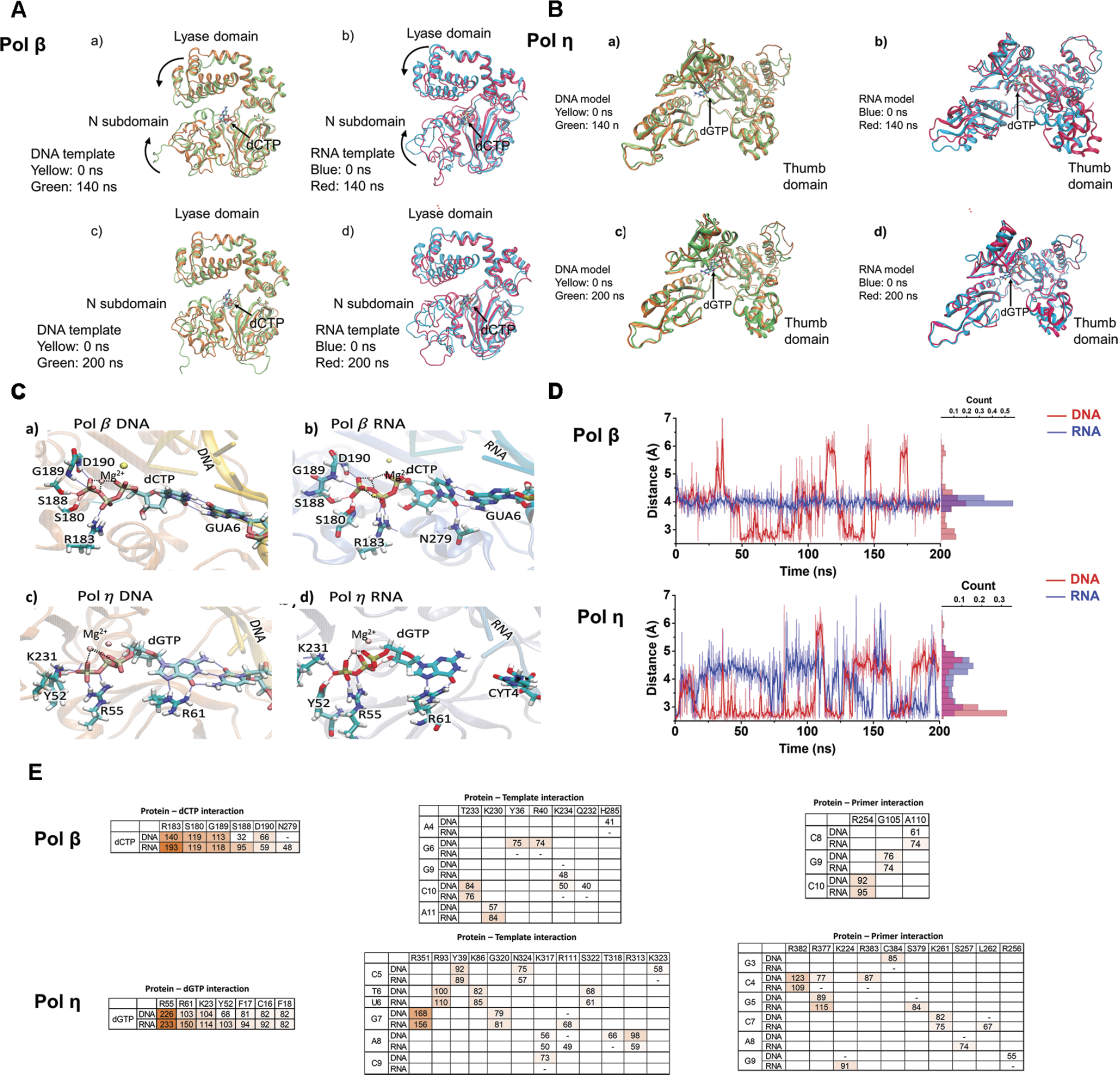
Molecular dynamics simulations of the ternary complexes of pol β and pol η with a 1 nt gap and open template substrate. (**A**) Molecular dynamics simulation of the ternary complex of pol β on the 1 nt gap substrates with a DNA (panel a and c) or RNA template (panel b and d) and incoming dCTP at 0 ns and 140 ns (panel a and b) and 0 ns and 200 ns (panel c and d) was presented. (**B**) Molecular dynamics simulation of the ternary complex of pol η on the open template substrates with a DNA (panel a and c) or RNA template (panel b and d) at 0 ns and 164 ns (panel a and b) and 0 ns and 200 ns (panel c and d) was presented. (**C**) The basepairing between dCTP and dG or G on the DNA (panel a and c) or RNA template (panel b and d) at 140 ns in pol β and 164 ns in pol η was presented. (**D**) Distance between the 3′-OH group of the DNA primer and α-phophosphate of the incoming nucleotide in pol β and pol η and histogram showed the distribution of distance change between the reacting oxygen atom in pol β and pol η with α-phosphate of the triphosphate with the presence of the DNA (red) or RNA template (blue). (**E**) The tables illustrate specific amino acids participating in H-bond interactions with dCTP, dGTP, DNA and RNA templates, and the DNA primer. The values indicate the hydrogen bond occupancy (%) determined using VMD. The % occupancy was calculated with a 3.5 Å distance cut-off and a 30º angle cut-off. The H-bond occupancy that exceeds 100 indicates that the same amino acid residue is involved in more than one H-bond at a time.

Employing the molecular dynamics simulation, we explored the dynamic interaction among pol β or pol η, a DNA primer, the incoming nucleotides, dCTP (pol β) or dGTP (pol η), and an RNA template (Figure 5). The molecular dynamics simulation during 200 ns revealed the dynamic structural change of the DNA polymerases in the presence of dCTP or dGTP at the catalytic center (Figure 5) (videos were deposited as the supplementary data at Zenodo). The results showed that pol β on the DNA (Figure 5A, panel a) or RNA template substrate (Figure 5A, panel b) adopted the closed conformation at 140 ns. The conformational change was accomplished by the closure movement between the dRP lyase and finger domain (Figure 5A, panel a, compare the domain in green with yellow, panel b, compare the domain in red with blue). The results further indicate that on the DNA template, the α-helixes on the dRP lyase domain of pol β moved toward its finger domain at 140 ns while the α helixes on the finger domain were also shifted toward the lyase domain (Figure 5A, panel a). In contrast, on the RNA template substrate, the α-helixes on the lyase domain and finger domain of pol β at 140 ns showed little change in their positions at 140 ns as compared with 0 ns suggesting a poor transition from the open to close conformation (Figure 5A, panel b). At 200 ns, the polymerase adopted the open conformation for both the DNA and RNA template substrates (Figure 5A, panel c and d). The conformation of pol η on an open DNA template with incoming dGTP exhibited little change during 0 ns, 140 ns, and 200 ns (Figure 5B, panels a-d) except that its thumb domain moved toward outside at 200 ns (Figure 5B, panel b) with an RNA template indicating that the enzyme adopted a steady open conformation. The results suggest that the polymerase exhibited more opened conformation to accommodate the RNA template. Further analysis on the basepairing between the incoming nucleotide with the template nucleotide on DNA or RNA showed that in the catalytic center of pol β, dCTP basepaired with the template dG and G (Figure 5C, panels a and b) at 140 ns. However, the triphosphate of dCTP on the RNA template substrate was completely disoriented compared with the DNA template (Figure 5C, panel b). In Pol η, dGTP failed to form a basepair with the template C on the RNA template at 164 ns (Figure 5C, panel d) although the nucleotide formed a stable H-bond with the template dC on the DNA. However, the orientation of the triphosphate of dGTP in the pol η ternary complex with the RNA template was almost identical with the DNA template (Figure 5C, compare panel c with panel d). We further demonstrated that the distance between the 3′-OH and α-phosphate of dCTP in the pol β ternary complex with the RNA template stayed at 4 Å during 200 ns (Figure 5D, top panel, blue line), whereas the distance with the DNA template exhibited a dynamic change between 1 and 7 Å (Figure 5D, top panel, red line). In contrast, the distance between the 3′-OH and α-phosphate of dGTP in the RNA-templated ternary complex of pol η predominantly stayed at 4.5 Å during the first 100 ns (Figure 5D, the bottom panel, blue line) but fluctuated between 1 Å and 6 Å in the second 100 ns overlapping with the dynamic change of the distance with the DNA template in the second 100 ns (Figure 5D, the bottom panel, red line). The H-bond occupancy analysis showed that pol β interacted with dCTP through R183, S180, and G189 with more than one H-bond (H-bond occupancy > 100) but with only one H-bond (H-bond occupancy < 100) through D190 on both the DNA and RNA template substrates (Figure 5E). Pol β interaction with the nucleotide at R183 and S188 was significantly increased on the RNA template. The polymerase also gained a new interaction with dCTP at N279 on the RNA template (Figure 5E). Pol η interaction with dGTP through R55, R61, K23, Y52, F17, C16 and F18 on the RNA template did not exhibit significant change compared with the DNA template. However, its nucleotide interaction via R61 and Y52 was significantly increased on the RNA template (Figure 5E). The results further indicated that pol β interaction with the RNA template through Y36, R40, K234, Q232 and H285 was lost compared with the DNA template. Its RNA interaction through T233 and K230 was not significantly changed. Moreover, it gained a new interaction on the RNA template through K234 (Figure 5E). In contrast, pol η showed little difference in interacting with the RNA and DNA template except that it lost its interaction with the RNA template through T318 and K323 and gained a new template interaction at R111 with A8 and G7 (Figure 5E). Pol β interaction with the primer did not exhibit a significant difference on the DNA and RNA template substrates (Figure 5E). Pol η lost its interaction with the DNA primer on the RNA template substrate at R377, R383, C384 and R256 but gained a new interaction with the primer at K224, S379, S257 and L262 (Figure 5E). The results suggest that pol β accommodated the RNA template to synthesize DNA by modulating its interactions with the incoming nucleotide and the RNA template. In contrast, pol η altered its interactions with the RNA template and DNA primer to synthesize DNA. In summary, our molecular dynamics simulation results revealed that the RNA template blocked the open-to-close conformational change of pol β and altered its interactions with the incoming nucleotide to accommodate the RNA template, thereby reducing pol β catalytic activity. On the other hand, pol η adopted an open conformation to accommodate the RNA template and alter its interactions with the DNA primer maintaining its efficient DNA synthesis. We further identified specific amino acids for pol β and pol η to interact with the incoming nucleotide, the RNA template, and the DNA primer.

### RNA-guided BER of a damaged base is accomplished through DNA nick translation

**Figure 6.**
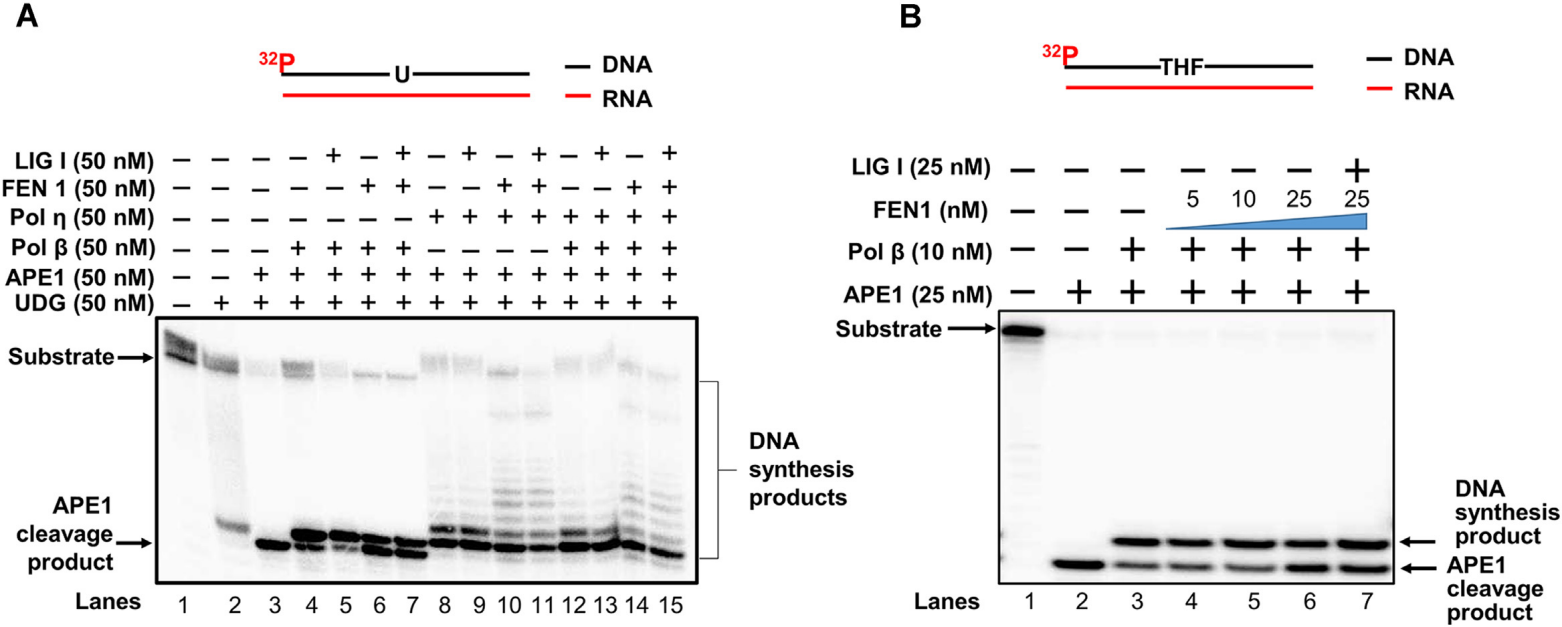
RNA-guided base damage repair by BER enzymes. (**A**) RNA-guided BER mediated by pol β and pol η was examined using the substrate containing an RNA template with an uracil on the DNA strand. Lane 1 represents substrate only. Lane 2 represents the reaction with the substrate and 50 nM of UDG. Lane 3 represents the reaction with the RNA template with 50 nM UDG and 50 nM APE1. Lane 4 represents RNA-templated DNA synthesis by 50 nM pol β in the presence of 50 nM UDG and 50 nM APE1. Lane 5 illustrates the reaction with 50 nM UDG and 50 nM APE1 in the presence of 50 nM of LIG I. Lanes 6–7 represent the reactions reconstituted with 50 nM UDG, 50 nM APE1, and 50 nM pol β in the presence of 50 nM FEN1 with the absence and presence of 50 nM LIG I. Lanes 8–9 illustrate RNA-guided BER reactions mediated by 50 nM of pol η in the presence of 50 nM UDG and 50 nM APE1 with the absence and presence of 50 nM LIG I. Lanes 10–11 represent pol η-mediated BER reactions reconstituted with 50 nM UDG, 50 nM APE1 and 50 nM pol η in the presence of 50 nM FEN1 with the absence and presence of 50 nM LIG I. Lanes 12–13 indicate the BER reactions reconstituted with 50 nM UDG, 50 nM APE1, 50 nM pol β, and 50 nM pol η in the absence and presence of 50 nM LIG I. Lanes 14–15 illustrate RNA-guided BER reconstituted with 50 nM UDG, 50 nM APE1, 50 nM pol η, 50 nM pol β and 50 nM FEN1 in the absence and presence of 50 nM LIG I. (**B**) RNA-guided BER was examined with the substrate containing an RNA template with a reduced AP site (THF residue) in the DNA strand. Lane 1 represents substrate only. Lane 2 indicates the RNA-guided 5′-incision of the reduced AP site by 25 nM APE1. Lane 3 illustrates RNA-templated DNA synthesis by 10 nM pol β. Lanes 4–6 represent the reactions reconstituted with 25 nM APE1, 10 nM pol β and increasing concentrations of FEN1 (5–25 nM). Lane 7 indicates RNA-guided BER reconstituted with 25 nM APE1, 10 nM pol β and 25 nM FEN1 in the presence of 25 nM LIG I. All experiments were performed in triplicate.

**Figure 7.**
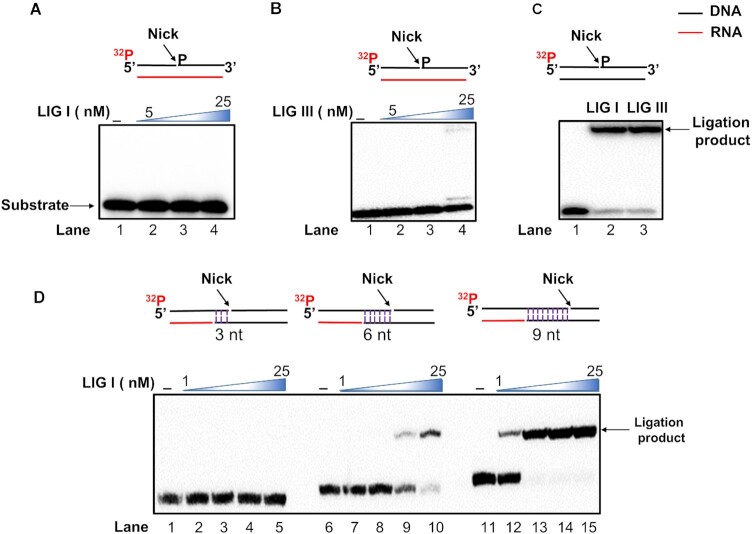
Ligation of RNA-templated nick by DNA ligases. (**A**) The RNA-templated nick substrate (25 nM) was incubated with increasing concentrations of LIG I at 37°C for 30 min. Lane 1 represents substrate only. Lanes 2–4 indicate the ligation reaction in the presence of LIG I (5 nM-25 nM). (**B**) The RNA-templated nick substrate (25 nM) was incubated with LIG III ranging from 5–25 nM at 37°C for 30 min. Lane 1 represents substrate only. Lanes 2–4 indicate the ligation reaction in the presence of LIG III (5 nM-25 nM). (**C**) DNA-templated nick ligation was examined by incubating the DNA nick substrate (25 nM) with 25 nM LIG I or 25 nM LIG III at 37°C for 30 min. Lane 1 represents substrate only. Lanes 2–3 represent the ligation reaction with LIG I and LIG III, respectively. (**D**) DNA ligation at a nicked DNA in the double-strand DNA region with various distances from the RNA template. The substrates with a DNA nick located at 3 nt, 6 nt and 9 nt from the RNA template (25 nM) were incubated with LIG I (1 nM-25 nM) at 37°C for 30 min. Lanes 2–5 illustrate the ligation reaction with the substrate containing a nick translocated 3 nt upstream to the RNA template. Lanes 7–10 represent the ligation with the substrate having a nick translocated 6 nt upstream to the RNA template. Lanes 12–15 represent the ligation reaction with the substrate containing a nick translocated 9 nt upstream to the RNA template. All experiments were performed in triplicate.

Our results further suggest that RNA-templated DNA gap-filling synthesis can lead to a unique BER pathway to repair DNA base damage that occurs on a DNA–RNA hybrid formed during DNA replication and gene transcription. To test this, we examined the RNA-guided repair of a DNA base lesion, an abasic site by reconstituting pol β- and pol η-mediated BER reactions using the substrates containing a native or reduced AP site (THF) with an RNA template (Figure 6). The results showed that the native AP site was incised by APE1 (Figure 6A, lane 3), leading to one nucleotide gap that was filled by pol β or pol η (Figure 6A, lanes 4 and 9). Pol β predominantly inserted one nucleotide to fill in the gap (Figure 6A, lanes 4–7). Pol η inserted multiple nucleotides to fill in the gap and displace the downstream strand (Figure 6A, lanes 8–11). Both pol β and pol η facilitated the gap-filling and strand-displacement synthesis (Figure 6A, lanes 12–15). We found that the presence of FEN1 inhibited pol β gap-filling synthesis (Figure 6A, lanes 6–7), whereas FEN1 stimulated the pol η DNA synthesis (Figure 6A, lanes 10–11 and 14–15). However, no repaired product was detected in reconstituted BER reactions with pol β or pol η or both pol β and pol η in the presence of LIG I (5 nM) (lane 5, 7, 9, 11, 13, and 15). We then tested pol β-mediated BER of a reduced AP site (THF residue) on the RNA template (Figure 6B). The results were the same as those from BER of a native AP site. APE1 incised the reduced AP site efficiently (Figure 6B, lane 2). Pol β then inserted one nucleotide to fill in the gap (Figure 6B, lane 3). The presence of FEN1 slightly inhibited pol β gap-filling synthesis (Figure 6B, lanes 4–6). No repair product was generated by LIG I (Figure 6B, lane 7). Since the completion of BER is accomplished by ligation of a nick, we then asked if the lack of the repair products resulted from the failure of nick ligation of the nick on the RNA template by a DNA ligase. We examined the ligation of a DNA nick on an RNA template by LIG I (Figure 7). We found that LIG I failed to seal the nick at 5–50 nM (Figure 7A, lanes 2–4). However, LIG IIIβ at 50 nM resulted in a small amount of repair product (Figure 7B, lane 4). Both LIG I and LIG III exhibited efficient ligation activity on the nick with a DNA template (Figure 7C, lanes 2–3). The results indicated that DNA ligases failed to seal the RNA-templated nick efficiently and generate the repaired product. We further asked if the nick must be translocated to a double-strand DNA region to be ligated by a DNA ligase. We tested this by examining the ligation of a nick located at different distances from the RNA template strand by LIG I. The results showed that LIG I failed to ligate a nick at 3 nt upstream of the RNA template (Figure 7D, lanes 2–5). LIG I at 1 nM and 5 nM also failed to ligate a nick 6 nt upstream of the RNA template. However, 10 nM and 25 nM LIG I sealed the nick resulting in a significant amount of the ligation product (Figure 7D, lanes 9–10). LIG I at 1 -25 nM generated the ligation product at the nick 9 nt upstream of the RNA template (Figure 7D, lanes 12–15). The results indicate that during RNA-guided BER, a DNA nick needs to be translocated at least 6 nt away from the RNA template for ligation by LIG I (Figure 7D, lanes 9–10). Since the DNA nick opposite the RNA template was only weakly ligated by LIG IIIβ (Figure 7B, lane 4), our results indicated that the RNA-guided BER was completed by the ligation of a nick that was translocated into a duplex DNA region.

## DISCUSSION

**Figure 8.**
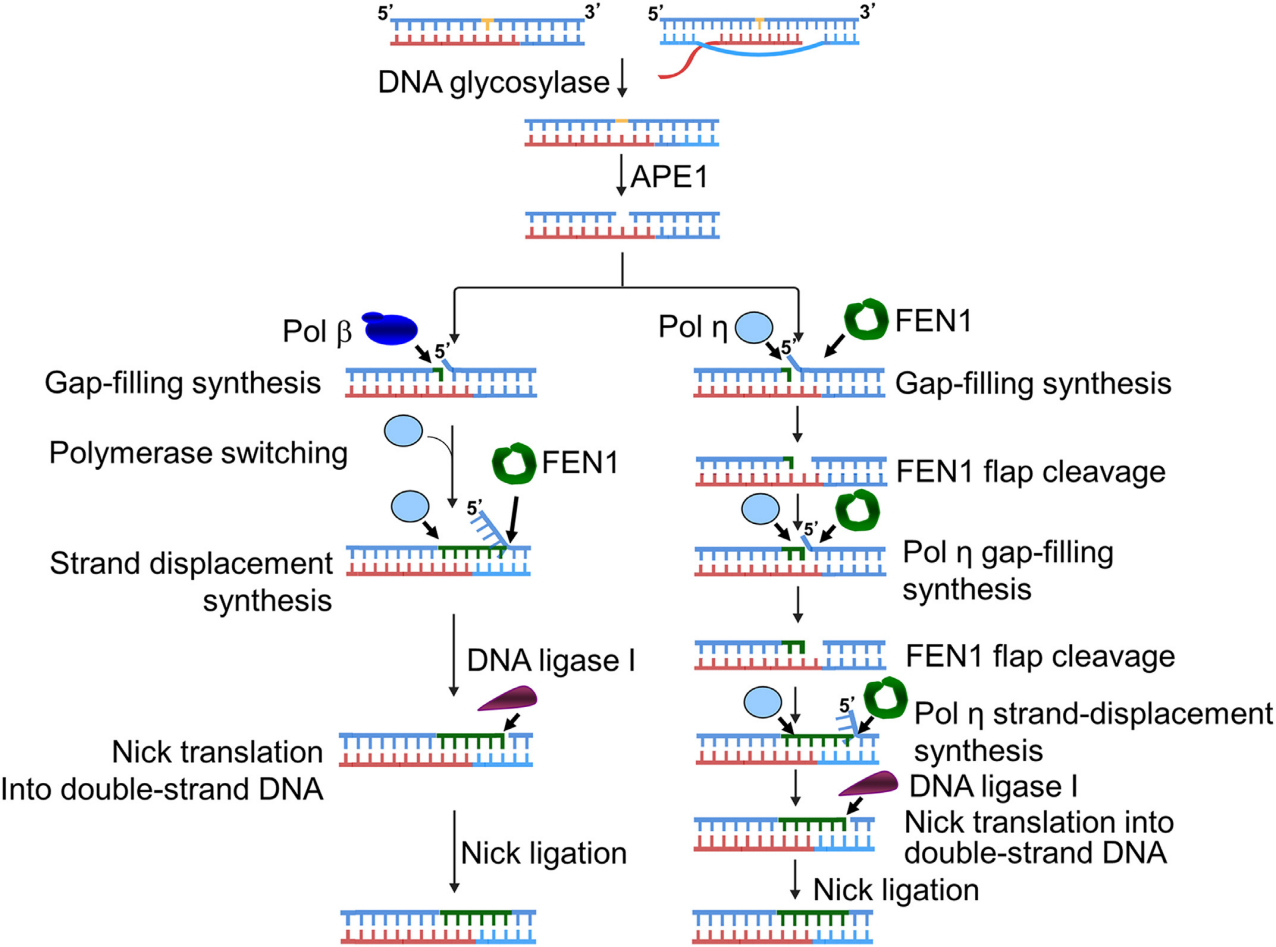
RNA-guided DNA base excision repair via nick translation. A DNA base lesion on template DNA of a DNA:RNA hybrid is removed by a DNA glycosylase leaving an abasic site that is subsequently incised by APE1 leaving 1 nt gap. Pol β fills in the gap, dissociates and switches with a translesion DNA polymerase such as pol η. The translesion DNA polymerase continues to perform RNA-templated strand-displacement synthesis translocating the nick into the DNA template regions and generating a nicked-flap. Subsequently, FEN1 cleaves the flap and generates a nick in DNA, which is sealed by DNA ligase I completing base damage repair (the subpathway on the left). Alternatively, the 1 nt gap can be directly captured by pol η. The polymerase can then perform the RNA-templated gap-filling synthesis and coordinate with FEN1 flap cleavage to translocate the nick into the double-strand DNA region. Pol η further performs strand-displacement synthesis to generate a nicked-flap that is removed by FEN1 cleavage. Subsequently, the nick is sealed by DNA ligase I completing the RNA-guided DNA base damage repair (the subpathway on the right). The graph was constructed with the assistance of the BioRender software with an license for publication authorized to the Biomolecular Sciences Institute (BSI) of Florida International University.

In this study, we characterized RNA-guided DNA synthesis by human DNA polymerases. We found that pol β, pol λ, and pol κ predominantly inserted one nucleotide on an RNA template, whereas pol η, pol θ, and pol ν performed multi-nucleotide DNA synthesis on an open RNA template and RNA-templated gap-filling and strand-displacement synthesis (Figures 1-4). We further demonstrated that pol β performed RNA-templated gap-filling synthesis with low efficiency (Table 1), and pol η performed gap-filling and strand-displacement synthesis with a higher efficiency than pol β (Figure 4A and 4C, Tables 1 and 2). We then demonstrated the RNA-templated removal of an uracil base lesion and the incision of an AP site by APE1 (Figure 6A, lanes 2–3 and Figure 6B, lane 2). We showed that pol η RNA-templated DNA synthesis was also stimulated by FEN1 (Figure 6A, compare lanes 10–11 and 14–15 with lanes 8–9 and 12–13) suggesting that pol η coordinated with FEN1 leading to the translation of the nick during RNA-guided BER. We found that LIG I and LIG IIIβ failed to efficiently ligate a DNA nick opposite to the RNA template efficiently (Figure 7A and 7B). The efficient ligation of a DNA nick only occurred in the regions of double-stranded DNA regions at least 6 nt away from the RNA template (Figure 7D). The results indicate that the gap-filling synthesis and strand-displacement synthesis by repair and translesion DNA polymerases can mediate RNA-guided BER by translocating the nick into a DNA-templated region via the coordination between the RNA-templated strand-displacement synthesis and FEN1 flap cleavage. Our results support a hypothetical model during which a DNA base lesion on the DNA template strand of a DNA–RNA hybrid is converted to 1 nt gap by a DNA glycosylase and APE1 (Figure 8). Pol β fills in the gap, switches with a translesion DNA polymerase such as pol η. pol η then performs strand-displacement synthesis leading to the formation of a nicked-flap that is subsequently cleaved by FEN1 (Figure 8, the subpathway on the left). The coordination between the polymerase and FEN1 leads to the translation of the nick in a DNA templated region. The nick is then sealed by LIG I completing the RNA-guided BER (Figure 8, the subpathway on the left). Alternatively, in a scenario where pol β gap-filling synthesis is inefficient, pol η can fill in the 1 nt gap creating a 5′-dRP flap that is removed by FEN1. This results in a gap that is filled in by pol η. The coordination between RNA-templated gap-filling synthesis by pol η and FEN1 cleavage leads to the translocation of the nick into a DNA-templated region. Subsequently, pol η performs strand-displacement synthesis to generate a nicked-flap within the DNA region. FEN1 then removes the flap leaving a nick for efficient ligation and completion of the RNA-guided BER (Figure 8, the subpathway on the right). It should be noted that using *in vitro* biochemical approaches and molecular dynamics simulation, we have revealed the molecular mechanisms underlying RNA-guided DNA base-damage repair illustrated in our hypothetical model (Figure 8). However, the model needs to be further validated *in vivo* in the future upon the development of new technologies for the determination of the RNA-templated DNA base damage repair capacity *in vivo*.

Here, for the first time, we have revealed the unique RNA-guided BER pathway that can be mediated by DNA polymerases through RNA-templated DNA nick translation. Our results demonstrated that the DNA nick had to be translated into a DNA-templated region for ligation (Figure 7D). We further demonstrated that the nick translation could be accomplished by the polymerase switch between pol β with pol η. Subsequently pol η performed the strand-displacement synthesis and coordinated with FEN1 flap cleavage to translate the nick to the DNA template region 6 nt away from the RNA template leading to nick sealing (Figure 8, the subpathway on the left). Alternatively, the nick translation could be mediated by the gap-filling synthesis and strand-displacement synthesis by the translesion DNA polymerases such as pol η in coordinating with FEN1 flap cleavage (Figure 8, the subpathway on the right) or by exploiting the ‘Hit and Run’ mechanism as proposed by Liu *et al.* (50). Thus, here we suggest a crucial role of nick translation in mediating the RNA-guided DNA base damage repair pathway.

Using steady-state kinetics, we found that the efficiency of RNA-templated gap-filling synthesis by pol β was reduced by 136 to 216-fold compared with the DNA-templated gap-filling synthesis (Table 1). In contrast, pol η exhibited the same efficiency in nucleotide incorporation on an RNA template as on a DNA template (Table 2). The catalytic efficiency of pol η for the gapped and open template substrates was 100-fold of that of pol β. Using molecular dynamics simulation, we then explored the underlying molecular basis of the RNA-templated DNA synthesis by pol β and pol η. We found that the RNA template disrupted the open-to-close conformational change of pol β (Figure 5A, pane b). Although the basepairing of the incoming dCTP with the G on the RNA template was maintained in pol β (Figure 5C, panel b), the triphosphate of dCTP was disoriented compared with the DNA template (Figure 5C, compared the orientation of the triphosphate of dCTP in panel b with panel a). The effects from the RNA template resulted in low efficiency of dCTP incorporation (Table 1). In contrast, although the dGTP:C basepair in pol η was disrupted by the RNA template (Figure 5C, panel d), the orientation of the triphosphate of dGTP with the RNA template was similar to the DNA template (Figure 5C, compare the triphosphate in panel c and with panel d). The results suggest that in pol η, an effective nucleophilic attack occurred between the 3′-OH group of the DNA primer and the α-phosphate of dGTP, leading to the formation of the phosphodiester bond and the high efficiency of catalysis (Table 2). Further analysis on the distance between the 3′-OH group of the DNA primer and the 5′-phosphate of the triphosphate showed that the RNA template resulted in the sustained 4Å distance between the 3′-OH and the α-phosphate of dCTP in the pol β–RNA–dCTP ternary complex, thereby leading to the failure of the nucleophilic attack by the 3′-OH group in pol β (Figure 5D, the top panel). On the other hand, with the RNA template, pol η managed to maintain similar dynamic changes in the distance between the 3′-OH and α-phosphate of dGTP to the DNA template in the second 100 ns (Figure 5D, the bottom panel). The results suggest that pol η adopted more flexible structures at its catalytic center to adapt the RNA template, thereby conferring its ability to accommodate the different configuration of ribonucleotides and tolerate bulky DNA lesions (51–54). Our results further suggest that the translesion DNA polymerases exploit the same structural basis for its DNA damage tolerance to perform RNA-templated DNA synthesis (55).

Using molecular dynamics simulation analysis, we have also revealed the dynamics of the conformational changes of pol β and pol η and their crucial roles in mediating RNA-templated DNA synthesis and DNA repair. We found that pol β adopted the typical open-to-close conformational change with the DNA template (Figure 5A). Surprisingly, we found that the pol β dRP lyase domain coordinated with the finger domain to exert the closed conformational change (Figure 5A). However, the RNA template inhibited the structural change by preventing the domains from moving toward each other (Figure 5A). These results are consistent with an early study from the Wilson group showing that the pol β 31 kDa polymerase domain alone exhibits significantly reduced DNA synthesis activity (56). Thus, our results demonstrate an essential structural and functional role of the dRP lyase domain in mediating the conformational change of pol β necessary for its DNA synthesis activity. Our results further indicate that pol β required the open-to-close conformational change to achieve its efficient catalysis. However, the polymerase ‘choked’ on the RNA–DNA hybrid with the A form structure, which blocked the conformational change necessary for efficient catalysis, resulting in the significantly reduced catalytic efficiency on the RNA template (Table 1). Interestingly, we found that pol η exhibited a relatively opened conformation with the presence of both the DNA and RNA template throughout the entire 200 ns simulation (Figure 5B). The RNA template induced the outward movement of the thumb domain of pol η at 164 ns simulation (Figure 5B, panel b) suggesting that the polymerase adopted a more opened conformation to accommodate the A form structure of the RNA–DNA hybrid achieving efficient catalysis. Similar to our findings, Chandramouly *et al.* have shown that pol θ also adopts an open conformation on the DNA–RNA and ddNTP ternary complex by rotating its finger domain outward and reconfiguring its thumb domain from α-helices into loops (23). All the results suggest that the translesion DNA polymerases adopt more opened conformation to accommodate the RNA template achieving efficient catalysis of nucleotide incorporation during DNA repair.

Herein, we also suggest that the catalysis of RNA-templated DNA synthesis is accomplished by the nucleophilic attack between the 3′-OH group of the DNA primer and 5′-phosphate of an incoming nucleotide rather than its basepair with an RNA template nucleotide. This notion is supported by our molecular dynamics simulation results showing that in pol β, although the incoming nucleotide formed a basepair with the base on the RNA template (Figure 5C, panel b), the triphosphate of the nucleotide adopted a distorted orientation (Figure 5C, compare panel b with panel b) that altered the dynamic change of the distance between the 3′-OH group and 5′-α phosphate (Figure 5D, top panel), thereby preventing the nucleophilic attack and leading to significantly reduced catalytic efficiency of pol β (Table 1). On the other hand, in pol η, although no solid basepair formed between the incoming nucleotide and the RNA template nucleotide, the DNA polymerase oriented the triphosphate of the incoming nucleotide in the similar orientation as the DNA template to align with the 3′-OH group (Figure 5C compare panel c with panel d), thereby maintaining the similar dynamic change of the distance between the two groups to the DNA template (Figure 5D, bottom panel) and leading to the similar catalytic efficiency to the DNA template (Table 2). The results are also consistent with our recent findings showing that pol η can incorporate a damaged nucleotide, cyclodeoxyadenosine by orientating the triphosphate to allow the nucleophilic attack of the 3′-OH group of a DNA primer to its α-phosphate (57). Our results further suggest that translesion DNA polymerases can exploit the nucleotide triphosphate orientation to perform RNA-templated nucleotide incorporation by sacrificing the basepairing and fidelity.

Our study suggests that RNAs, the transcripts of DNA can play an active role in guiding the repair of DNA base damage by exploiting RNA–DNA hybrids formed in highly transcribable genes to maintain genome stability and integrity. The fact that translesion DNA polymerases performed RNA-templated DNA synthesis on the HCV and COVID-19 genomic RNA sequences (Supplementary Figure S1) further suggests a potential role of translesion DNA polymerases in mediating viral reverse transcription, bypass of RNA base lesions, and cDNA mutations during viral infection. This notion is also supported by the findings from the Guengerich group (31,32). Furthermore, using molecular dynamics simulation, we revealed a unique molecular mechanism underlying RNA-guided DNA base damage repair. Thus, our studies will further facilitate the discovery of new targets for RNA-based therapy, prevention, and diagnosis of cancer and viral infection.

Our results showed that the replicative DNA polymerase, pol δ failed to perform RNA-templated DNA synthesis (Figure 1, lane 2). In contrast, repair and translesion DNA polymerases, pol β, pol κ, pol η, pol ν and pol θ, except pol ι exhibited DNA synthesis by inserting 1–10 nt (indicated by red circles) on the RNA open template and 1 nt-gapped substrates (Figures 1, 2A, 3A, 4A and 4C). The results further suggest that the repair and translesion DNA polymerases rather than replicative DNA polymerases can employ an RNA template to synthesize a short patch of DNA fragments and mediate the efficient repair of DNA base lesions. However, it is possible that the processive RNA-templated DNA synthesis may be accomplished via the interaction between the repair and translesion DNA polymerases with their cofactors. This may result in the synthesis of larger DNA fragments leading to DNA homology-mediated end-joining for double-strand DNA break repair.

It should also be noted that recent studies have shown DNA repair and translesion polymerases can incorporate ribonucleotides in DNA. Thus, it is possible that RNA-templated incorporation of a ribonucleotide during BER may occur, considering the concentration of ribonucleotides triphosphate (rNTPs) are much higher than deoxyribonucleotide. Since it is reported that pol β and pol η exhibit about 8000- and 3000-fold lower efficiency to incorporate rNTPs than dNTPs on a DNA template (30,58), it is likely that RNA-templated incorporation of rNTPs by pol β and pol η is much less efficient than their DNA-templated rNTP incorporation. It is noteworthy to further explore the RNA-guided ribonucleotide incorporation in DNA and its fidelity in the future.

Interestingly, we found that M-MLV RT failed to synthesize DNA to the end of the RNA template. This could result from the secondary structures formed in the RNA template. Using the RNAstructure software developed by the Mathews Laboratory (59) and the OligoAnalyzer Tool from Integrated DNA Technology Inc (IDT), we analyzed the formation of the possible secondary structures on the unannealed region of the RNA template and their stability. The RNAstructure software showed that no secondary structures could be predicted in the region. The IDT OligoAnalyzer Tool predicted several secondary structures with the predicted ΔG of 1.6–1.8 kcal mol^−1^ (Supplementary Figure S3) and *T*_m_ of –11.5°C to –2.3°C. The results indicated that no secondary structures in the unannealed region of the RNA template were generated under our experimental condition at 37°C for RT to synthesize DNA. Based on the fact that the M-MLV RT exhibits less processive DNA synthesis activity and tends to pause during its DNA synthesis (60), it is likely that under our experimental condition, the RT pausing led to inefficient DNA synthesis. Since our results showed that RT performed efficient DNA synthesis on the DNA template (Figure 1, lane 24), it is possible that the RT paused by the unique conformation of the ribose such as sugar puckering during RNA-templated DNA synthesis.

## DATA AVAILABILITY

The videos of the molecular dynamics simulations of the DNA polymerases were deposited into Zenodo linked with ORCID to enhance the public accessibility of the results. A Zenodo personal access token for Yuan Liu was created for the deposit.

## Supplementary Material

gkac1178_Supplemental_Files
