# Supplementary material for: RNA-guided DNA base damage repair via DNA polymerase-mediated nick translation
Source: Nucleic Acids Res. 2022 Dec 19;51(1):166–81. doi: 10.1093/nar/gkac1178 (PMC9841414; doi:10.1093/nar/gkac1178)
Supplement: gkac1178_Supplemental_Files [file gkac1178_supplemental_files.zip › Supplementary Table, Figures and Figure Legends.pdf]

Supplementary Table S1

Oligo nucleotides[a]	nt	Sequence (5'-3')
Upstream Strand		
U1	19	CTTTCCTTTTACGTCATCC
Downstream Strands		
D1	16	pGGGGCAGACTGGGTGG
D2	16	pFGGGGCAGACTGGGTGG
D3	17	pGGGGGCAGACTGGGTGG
Template Strands		
T1	36	CCACCCAGUCUGCCCC**C**GGAUGACGUAAAAGGAAAG
T2 (HCV)	30	GUGGUACUGCCUGAUAGGGUGCUUGCGAGU
T3 (COVID-19)	30	GGUGUUGGUUACCAACCAUACAGAGUAGUA
T4 (DNA template	36	CCACCCAGTCTGCCCC**C**GGATGACGAAAAGGAAAG

[a] The nucleotide opposite to the 1 nt gap is on boldface. “F” indicates tetrahydrofuran. “p” represents a phosphate group.

Supplementary Figure S1

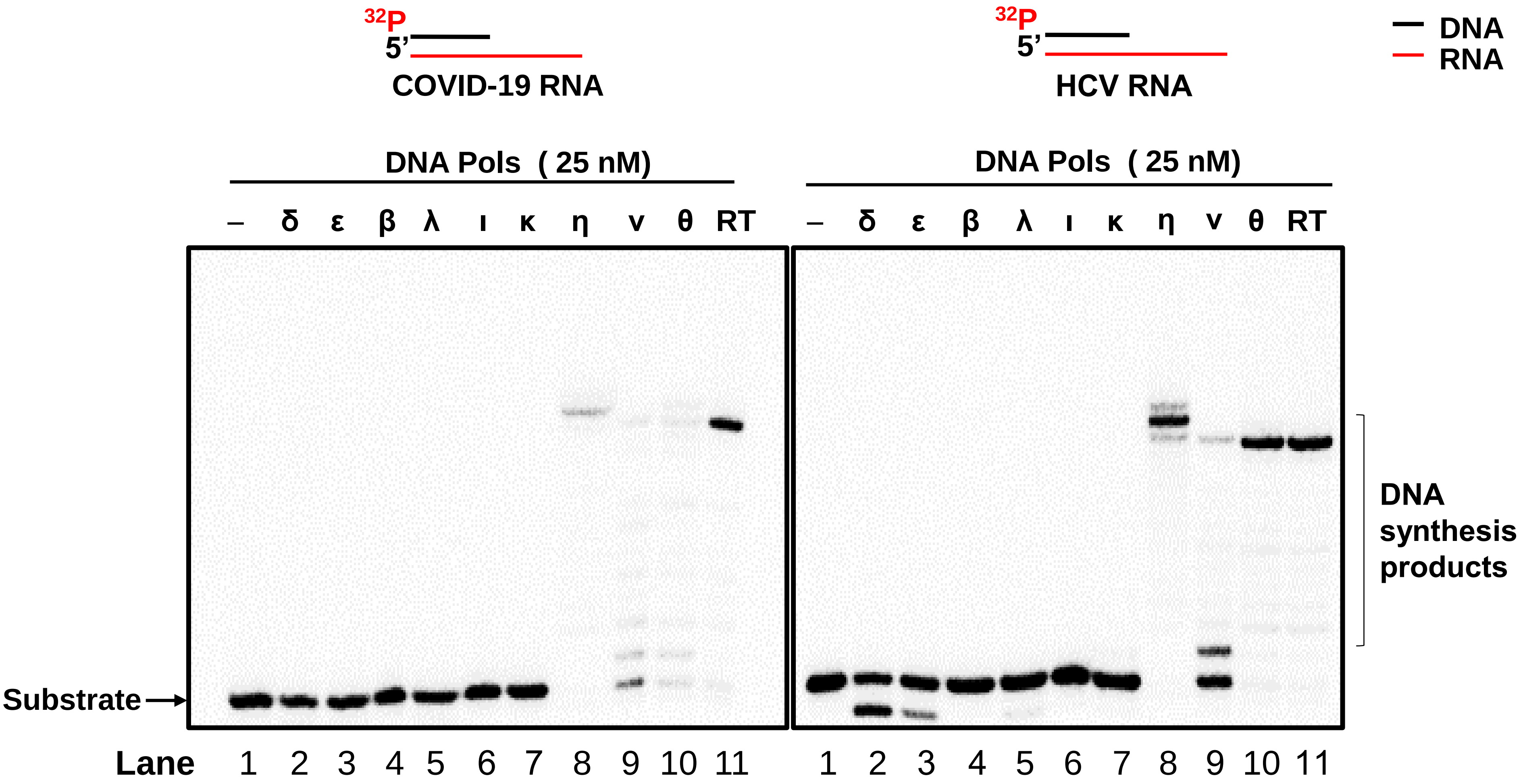

Supplementary Figure S2

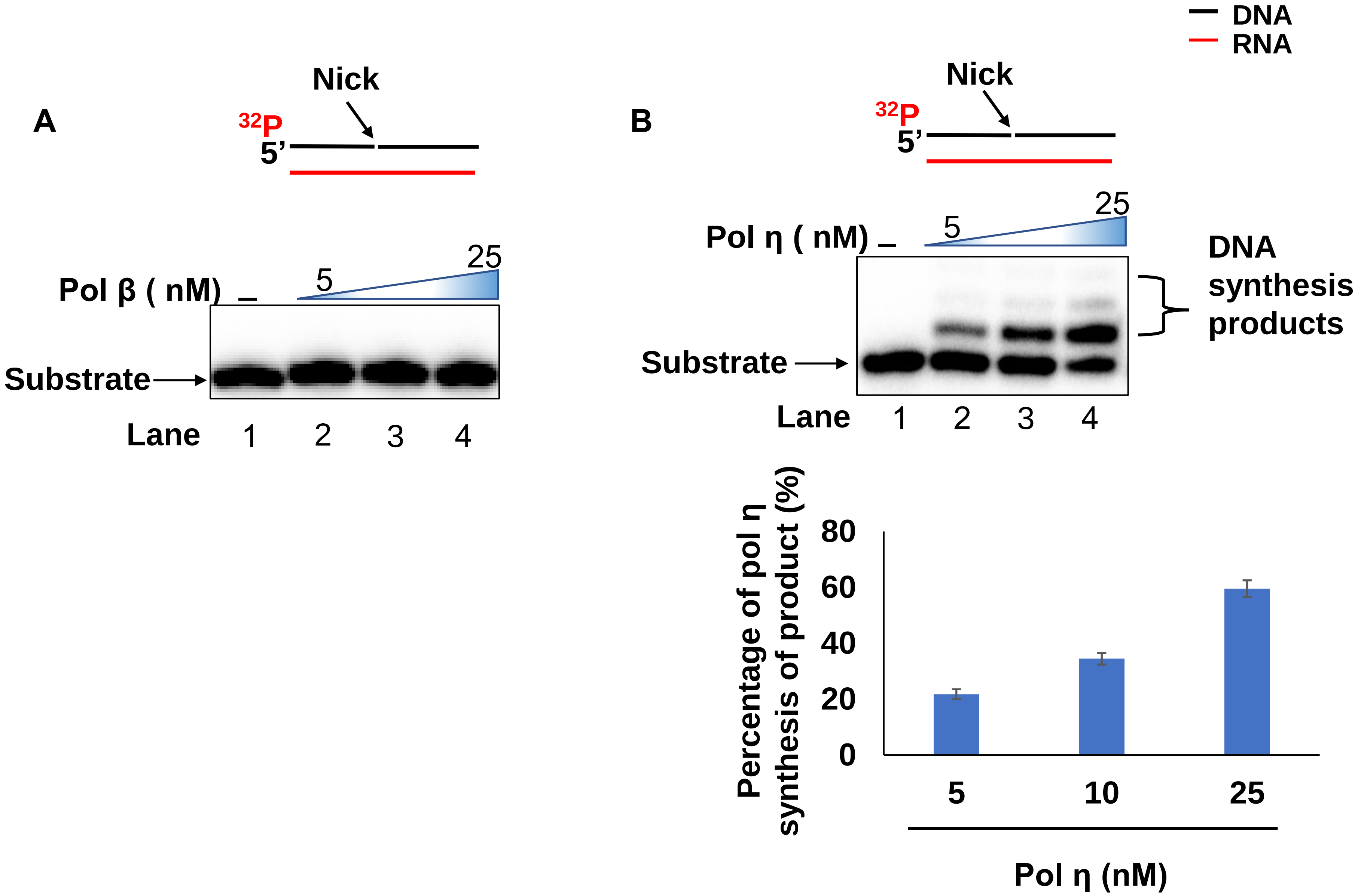

Supplementary Figure S3

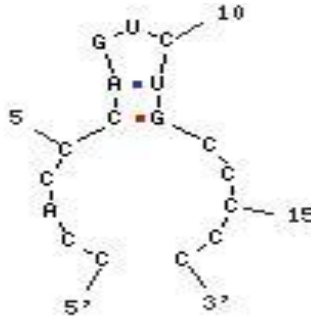

1

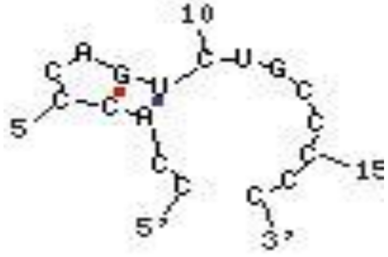

2

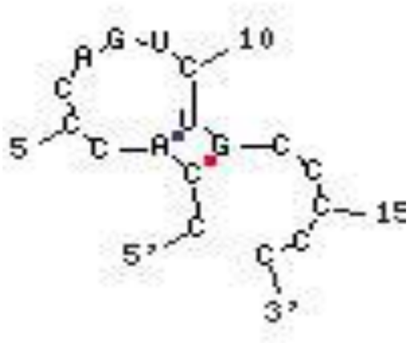

3

Structure	ΔG (kcal.mole^{-1})	T_m (^oC)	ΔH (kcal.mole^{-1})	ΔS (cal.K^{-1}mole^{-1})
1	1.6	-5.8	-10	-37.4
2	1.6	-11.2	-8.7	-33.21
3	1.8	-2.3	-12.4	-45.78

Supplementary Figure Legend

Supplementary Figure S1. DNA synthesis is guided by the COVID-19 and HCV RNA template. The DNA synthesis guided by the COVID-19 and HCV RNA template was examined in the presence of 25 nM RNA-DNA open template substrate and 25 nM pol δ, pol ε, pol β, pol λ, pol ι, pol κ, pol η, pol ν, pol θ, and reverse transcriptase (RT). Lane 1 represents substrate alone. Lanes 2-11 represent the DNA synthesis reaction with pol δ, pol ε, pol β, pol λ, pol ι, pol κ, pol η, pol ν, pol θ, and RT, respectively. The experiments were conducted in triplicate.

Supplementary Figure S2. **RNA-templated DNA synthesis by pol β and pol η at a nicked DNA**. RNA-templated DNA synthesis by pol β and pol η was determined in the presence of 25 nM nicked substrate. (A) RNA-templated DNA synthesis by various concentrations of pol β (5 nM, 10 nM, and 25 nM). (B) RNA-templated DNA synthesis by pol η (5 nM, 10 nM, and 25 nM). The quantification of the DNA synthesis products is illustrated below the gel. Lane 1 represents the substrate alone. Lanes 2-4 represent the DNA synthesis reactions in the presence of different concentrations of DNA polymerases (5-15 nM).

Supplementary Figure S3. The prediction of the secondary structure formation in the unannealed region of the RNA template. The predicted secondary structures in the unannealed region of the RNA template were analyzed by the OligoAnalyzer Tool from Integrated DNA Technology Inc (IDT). The prediction was performed under the condition that mimics our experimental condition in the presence of 50 mM NaCl, 5 mM Mg^{2+}, 25 nM RNA template, 50 μM dNTPs at 37°C.
